# Supplementary material for: Anomalous enhancement of charge density wave in kagome superconductor CsV3Sb5 approaching the 2D limit
Source: Nat Commun. 2023 Apr 29;14:2492. doi: 10.1038/s41467-023-38257-3 (PMC10148882; doi:10.1038/s41467-023-38257-3)
Supplement: Supplementary file 1 — Supplementary Information [file 41467_2023_38257_MOESM1_ESM.pdf]

Supplementary Information for "Anomalous enhancement of charge density wave in kagome superconductor CsV_3Sb_5 approaching the 2D limit"

Boqin Song[1,*], Tianping Ying[2,*✉], Xianxin Wu[3,*], Wei Xia[4,5,*], Qiangwei Yin[6,*], Qinghua Zhang[2], Yanpeng Song[2], Xiaofan Yang[1], Jiangang Guo[2], Lin Gu[2], Xiaolong Chen[2], Jiangping Hu[2], Andreas P. Schnyder[7], Hechang Lei[6✉], Yanfeng Guo[4✉], and Shiyan Li[1,8,9✉]

[1]*State Key Laboratory of Surface Physics, Department of Physics, Fudan University, Shanghai 200433, China*

[2]*Beijing National Laboratory for Condensed Matter Physics, Institute of Physics, Chinese Academy of Sciences, Beijing 100190, China*

[3]*CAS Key Laboratory of Theoretical Physics, Institute of Theoretical Physics, Chinese Academy of Sciences, Beijing 100190, China*

[4]*School of Physical Science and Technology, ShanghaiTech University, Shanghai 201210, China*

[5]*ShanghaiTech Laboratory for Topological Physics, Shanghai 201210, China*

[6]*Laboratory for Neutron Scattering, and Beijing Key Laboratory of Optoelectronic Functional Materials MicroNano Devices, Department of Physics, Renmin University of China, Beijing 100872, China*

[7]*Max-Planck-Institut für Festkörperforschung, Heisenbergstrasse 1, D-70569 Stuttgart, Germany*

[8]*Collaborative Innovation Center of Advanced Microstructures, Nanjing 210093, China.*

[9]*Shanghai Research Center for Quantum Sciences, Shanghai 201315, China*

Supplementary Note 1: TEM image of the transverse section of CsV_3Sb_5 thin flakes

To answer the question of whether the V_3Sb_5 kagome layer can survive when approaching the atomic limit, we employ the STEM to check the crystallinity in the atomic-thin flakes. We find the edge area will naturally curl to expose its transverse section. Supplementary Fig. 1 shows the morphology of the edge area. It is determined to be a 2L flake from the length (white lines) considering the lattice parameter c = 9.8 Å. Thus, we can use its intensity contrast as a gauge to determine the thickness of other thin flakes.

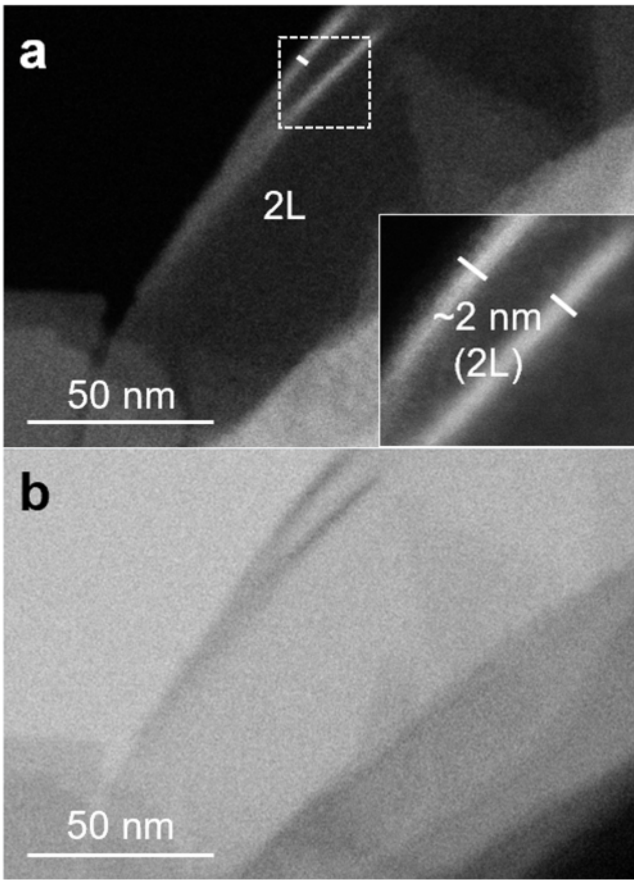

Supplementary Figure 1 | Transverse section of CsV_3Sb_5 atomic thin flakes. a, b Low-magnification ADF (**a**) and ABF (**b**) STEM images of the edge of the exfoliated CsV_3Sb_5 thin flake. The short white lines are the transverse section of the curled edge part.

Supplementary Note 2: Thickness determination in TEM

Supplementary Fig. 2a illustrates the low magnification STEM image of an exfoliated sample with a stair structure. With the intensity contrast gauge obtained in Supplementary Note 1, we can determine the thickness of each step of the stair from the ADF intensity line profile plotted in Supplementary Fig. 2b, c. A few flat areas with certain thicknesses can be selected for SAED and ABF-STEM measurements (see main text Fig. 1c, d).

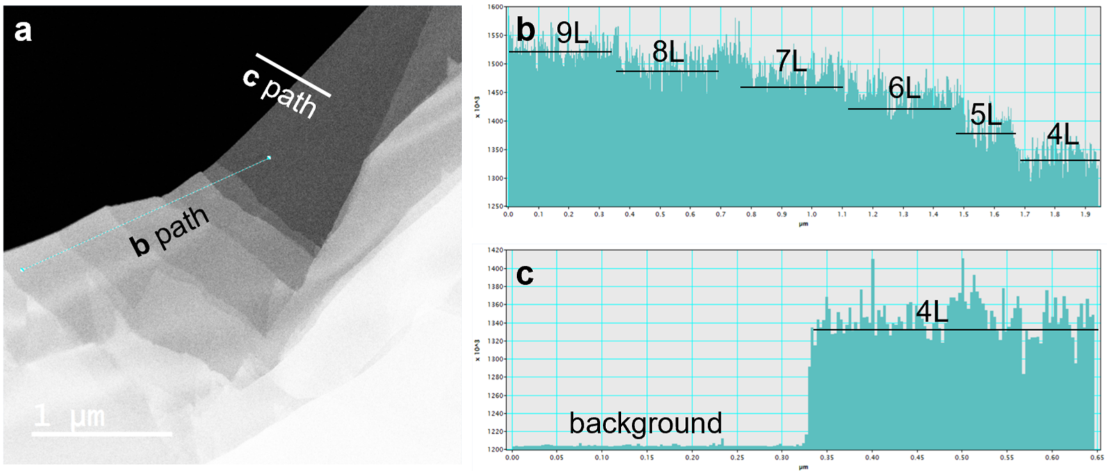

Supplementary Figure 2 | Determination of the flake thickness. a Low-Magnification STEM image of the exfoliated flakes. **b, c** Line profile of ADF intensity along different paths shown in **a**.

Supplementary Note 3: Quantitative analysis of the exfoliated thin flakes

STM studies found a fully occupied Cs surface layer can be unstable, often leading to randomly distributed Cs atoms that prone to clustering[1]. Loss of surface Cs distinctly manipulate the VHS relative to the Fermi surface, especially in the thin flakes[2]. On the other hand, Cs layers bond the V_3Sb_5 layers together, preventing interlayer slide. Therefore, it is interesting to investigate the stability of Cs layers

between the V_3Sb_5 kagome layers in these free-standing thin flakes. Supplementary Fig. 3c, d show the HAADF-STEM image of an 8L flake along the *c* axis. The clear atomic resolution image negates the possibility of interlayer slide. Hence, we can use the primary crystal structure to calculate the number of the elements, as shown in Supplementary Fig. 3a. Intensity lines of the marked region are plotted in Supplementary Fig. 3e, f. The integrated intensity is proportional to Z^2, where Z is the atomic number of the elements. Comparing the intensity with atomic number, a model of 8L sample without the outmost Cs layers corresponds best, as shown in Supplementary Fig. 3b.

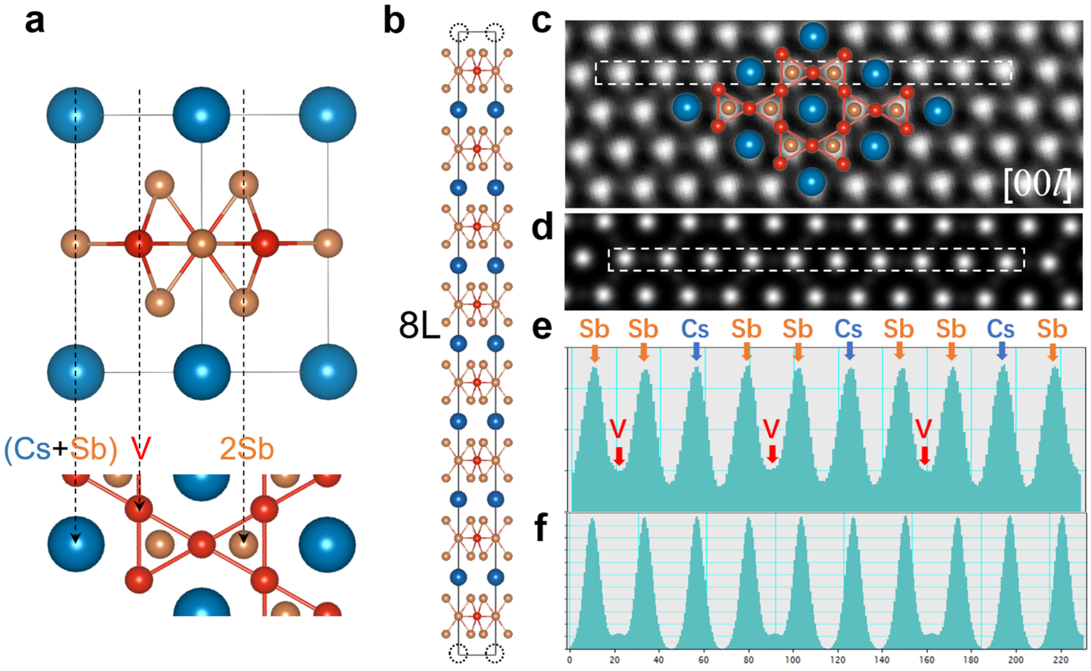

Supplementary Figure 3 | Quantitative analysis of the 8L sample. a Side view of the CsV_3Sb_5 structure along the [110] direction. Dotted arrows indicate the direction of the projection along the *c* axis. **b** Schematic structure of an 8L sample. Open circles on the out-most of the sample illustrate the loss of surface Cs. **c** Experimental high-angle annular dark-field scanning transmission electron microscopy (HAADF-STEM) image of an 8L sample. **d** Simulated atomic resolution image, based on the crystal structure model shown in **b**, was generated using the xHREM^{TM} software. **e** Intensity line profile of the region marked by the dotted line shown in **c**. **f** Simulated intensity

of the 8L sample.

Supplementary Note 4: Thickness determination

Supplementary Fig. 4a displays an typical optical image of few-layer CsV_3Sb_5 flakes on Al_2O_3 film on a sapphire substrate. We determined the layer number of the flakes with an atomic force microscope (AFM) and optical contrast. Here the optical contrast is transmittance defined as $G^{\mathrm{T}}_{\mathrm{sample}}/G^{\mathrm{T}}_{\mathrm{substrate}}$, where $G^{\mathrm{T}}_{\mathrm{sample}}$ and $G^{\mathrm{T}}_{\mathrm{substrate}}$ are the intensity of the transmission (T) through the sample and substrate, respectively, in blue channel of the image captured with a charge-coupled device (CCD) camera. The transmittance of various numbers of layers follows the Beer-Lambert Law (Supplementary Fig. 4b), which enables us to determine the layer number quickly and precisely. After that, we deposit Cr/Au contacts through a stencil mask on the thin flakes for transport measurements (Supplementary Fig. 4c, d).

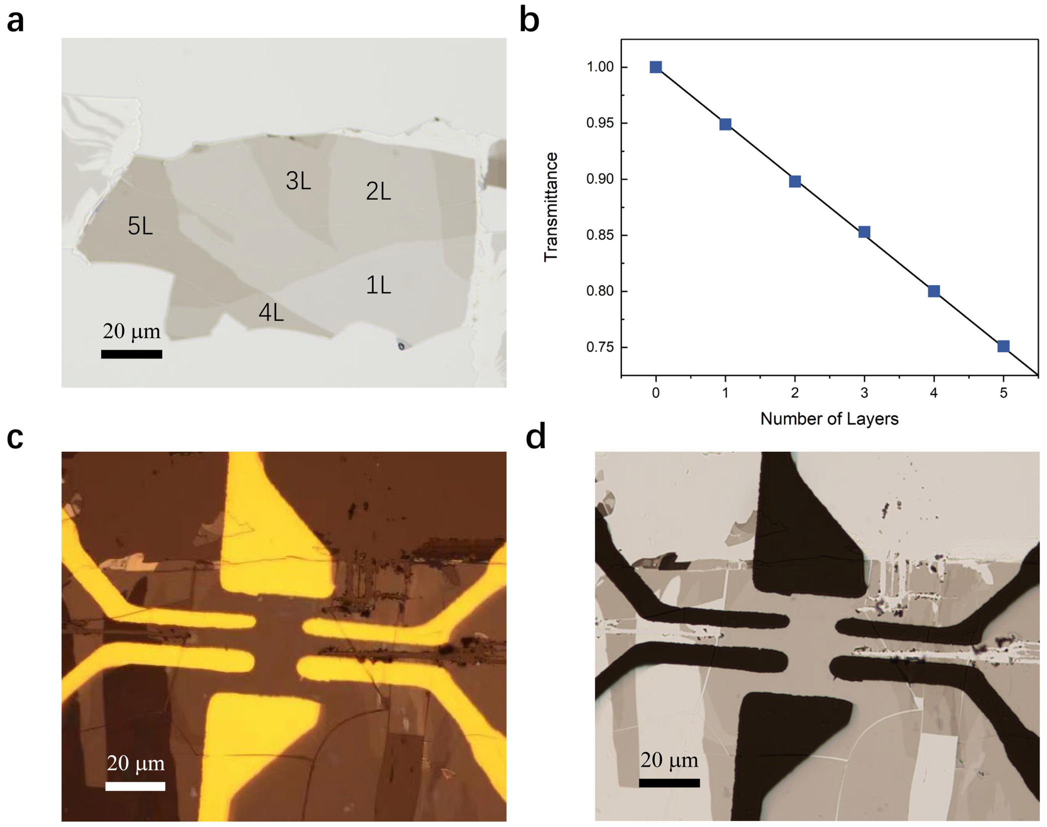

Supplementary Figure 4 | CsV_3Sb_5 thin-flake device fabrication. a Optical image

of few-layer flakes of CsV_3Sb_5 cleaved onto thermally evaporated Al_2O_3 thin film (thickness ~70 nm). The CsV_3Sb_5/Al_2O_3 stack is supported on a sapphire substrate. Image was taken in transmission mode. Number of layers is labeled on the selected areas with the scale bar of 20 μm. **b** Transmittance as a function of the number of layers. The transmittance (filled squares) follows the Beer-Lambert law (solid line). **c**, **d** Optical image of a device in reflection and transmission mode, respectively. Metal (Cr/Au) contacts to the sample were thermally evaporated through a stencil mask. Excess CsV_3Sb_5 flake that shorts adjacent electrodes are removed with a sharp tip.

Supplementary Note 5: Stability of the thin-flakes device

To eliminate the influence of remnant oxygen in glove box and heating degradation (thermal release) towards sample quality during our fabrication process, we examined the stability of the devices. Supplementary Fig. 5a shows the normalized resistance of sample #37 just fabricated (blue line) and stored in a glove box for 40 days (orange line). Despite the slope becoming a little gentler below T_{CDW}, the peak of the derivative maintains its position (Supplementary Fig. 5b), indicating adequate stability for fabrication process (3 hours generally). Thermal stability is tested on sample #39. The slope of normalized resistance and the T_{CDW} remains the same before and after heating at 120 °C (used in thermal release) in a high vacuum chamber (Supplementary Fig. 5c, d), revealing excellent stability of the fabricated thin-flake devices against thermal fluctuations.

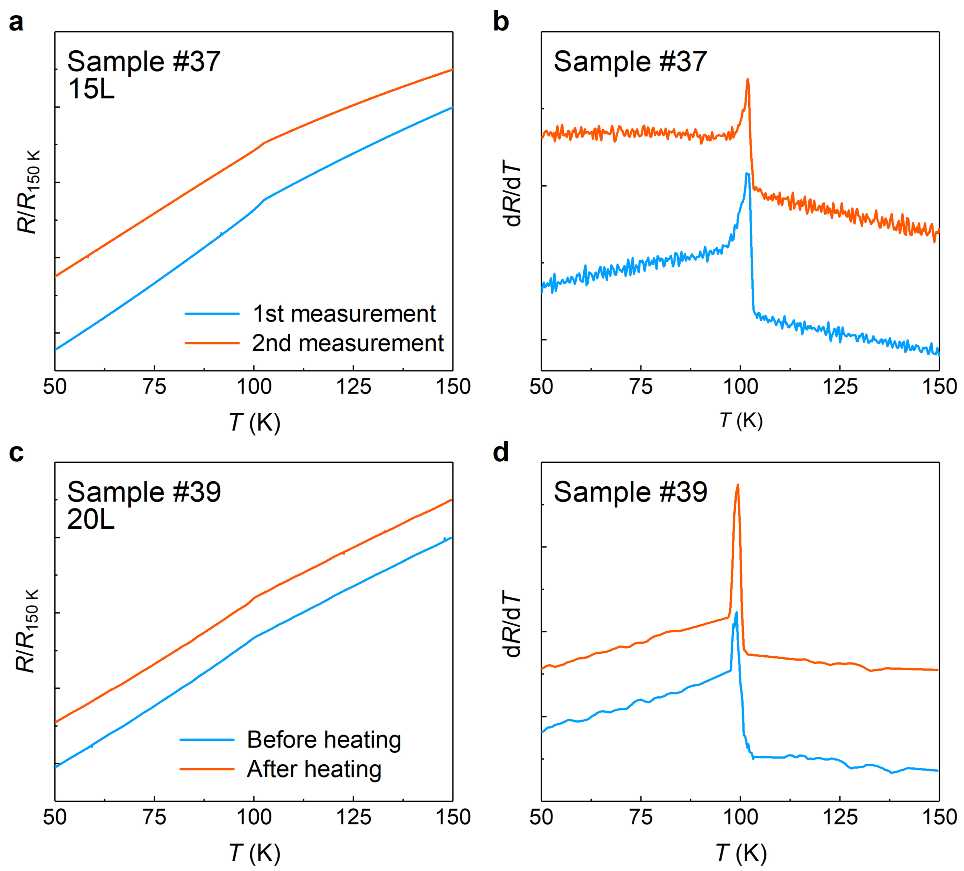

Supplementary Figure 5 | Stability of the thin-flake devices. a A 15L sample measured immediately after the nano fabrication (1st) and 40 days stored in a glove box (2nd). **c** A 20L sample measured before and after heating at 120 °C for half an hour in a high vacuum chamber. **b**, **d** Derivation of resistance in **a** and **c**. The highly reproducible kink temperature illustrates the stability of the fabricated devices. Lines in **a-d** are all shifted vertically for clarity.

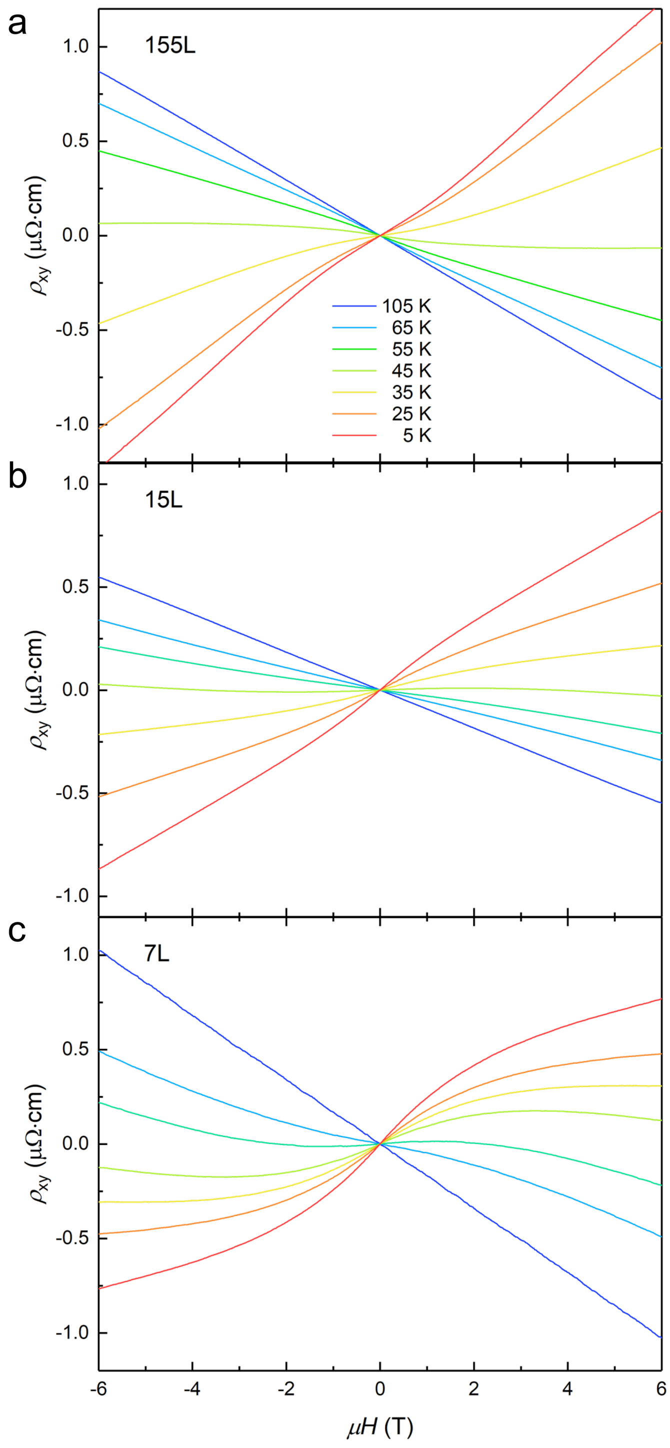

Supplementary Figure 6 | Temperature dependence of Hall resistivity. a-c Temperature-dependent Hall resistivity of 155L, 15L and 7L samples, respectively. The skew resulting from anomalous Hall effect is located at low fields, while the normal Hall resistivity is linear at high magnetic fields in all sample. The normal Hall coefficient change sign as temperature increase to around 40 K. Meanwhile, the AHE remains to exist to higher temperatures.

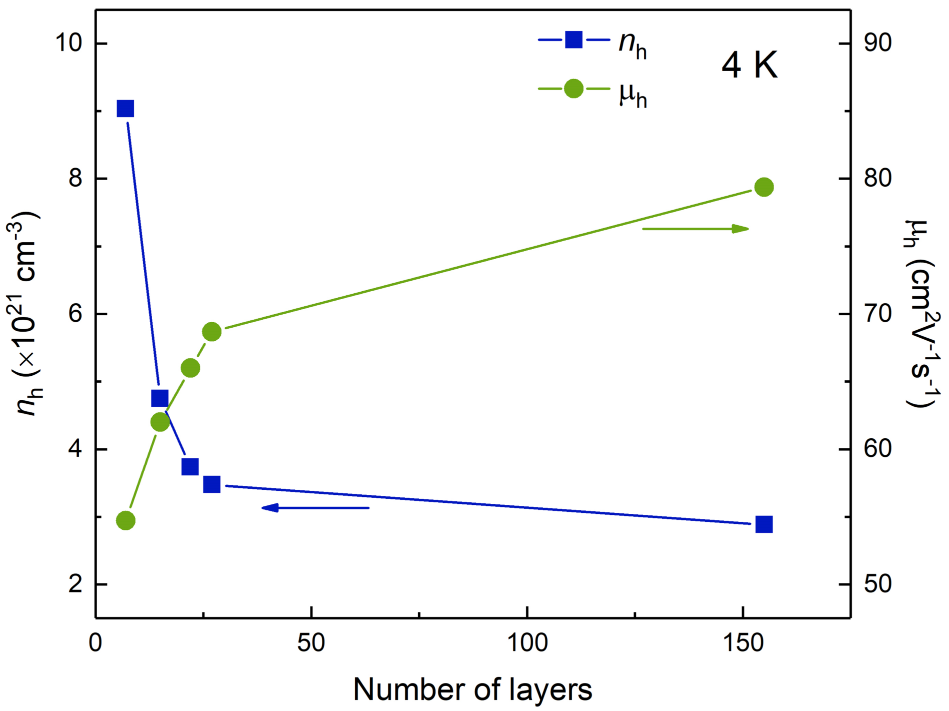

Supplementary Figure 7 | Carrier density and mobility at 4 K. Carrier density and mobility of CsV_3Sb_5 samples with various thickness extracted from high field Hall data at 4 K. Hole density increase with the reduction of thickness.

Supplementary Note 6: Hall measurement and anomalous Hall extraction

Supplementary Fig. 8a-e sketch Hall resistivity of CsV_3Sb_5 flakes with various thicknesses. It can be roughly seen that the anomalous Hall resistivity decreases to negligible and show a sign change with the reduction of sample thickness. However, the normal Hall at high-field does not change its sign. To clearly illustrate the anomalous Hall effect in CsV_3Sb_5 thin flakes, we extract the anomalous Hall term from the ρ_{xy} by subtracting the local linear term and plot them in Fig. 2d of the main text. To further confirm the AHE nature of sign-changed data of thin flakes, we carry out an angle-dependent measurement in a 7L sample. Supplementary Fig. 8f shows the ρ_{xy} varied with the angle θ between the magnetic field and the c axis.

Supplementary Fig. 8g, e show the ρ^{AHE} dependence on θ and cos(θ), respectively. The AHC is angle independent until magnetic field is tiled away from the *c* axis by about 30°, then it rapidly decreases to 0. ρ^{AHE} cannot be scale linearly with the out-of-plane component of magnetic field (cos(θ)), confirming the AHE origin of the skewed Hall resistivity at low field in atomically thin flakes and the AHE extraction is correct.

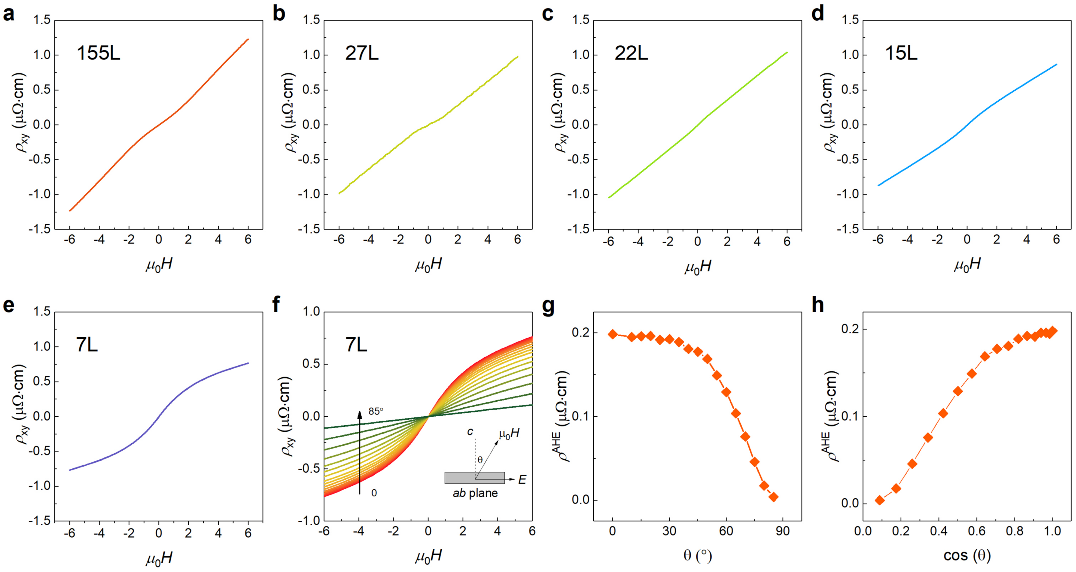

Supplementary Figure 8 | Hall measurement of various CsV_3Sb_5 flakes. a-e Raw Hall data of Fig. 2d in main text. **f** Hall resistivity as the variation of angle θ between magnetic field and *c* axis. The inset is a schematic configuration of magnetic field relative to the applied electrical field. All ρ_{xy} in **a-f** are measured at 5 K. **g**, **e** Extracted Anomalous Hall resistivity against θ and the out-of-plane component of magnetic field (cos(θ)), respectively.

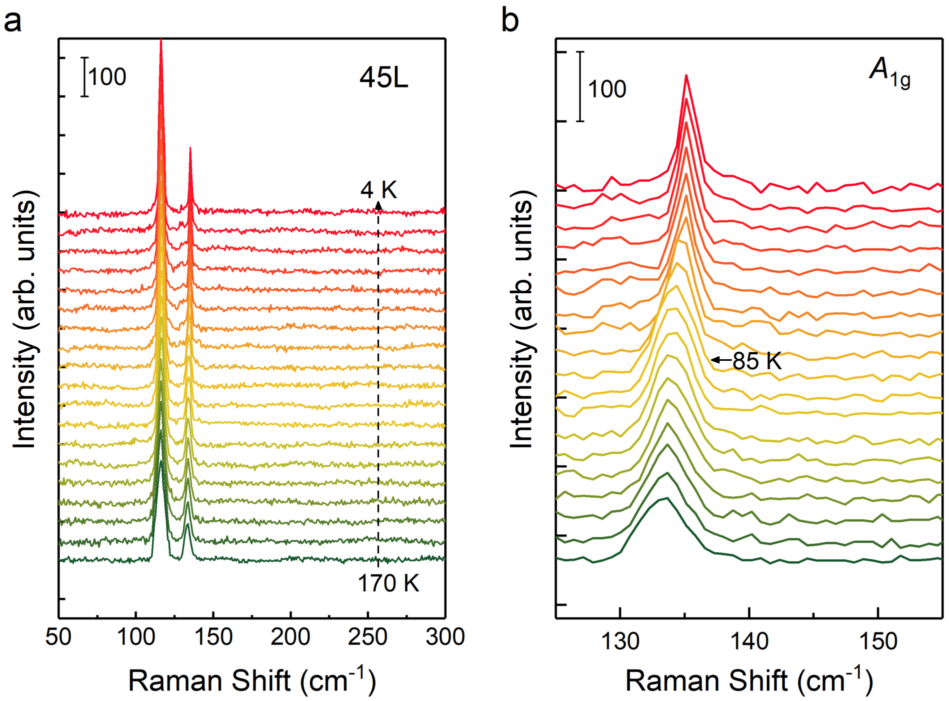

Supplementary Figure 9 | Temperature dependent Raman spectrum of 45L sample. a The Raman spectrum for 45L sample at temperature varied from 170 K to 4 K. Only two main lattice peak emerge. b Detail of evolution of the A_{1g} mode. Below 85 K, the A_{1g} mode hardens sharply.

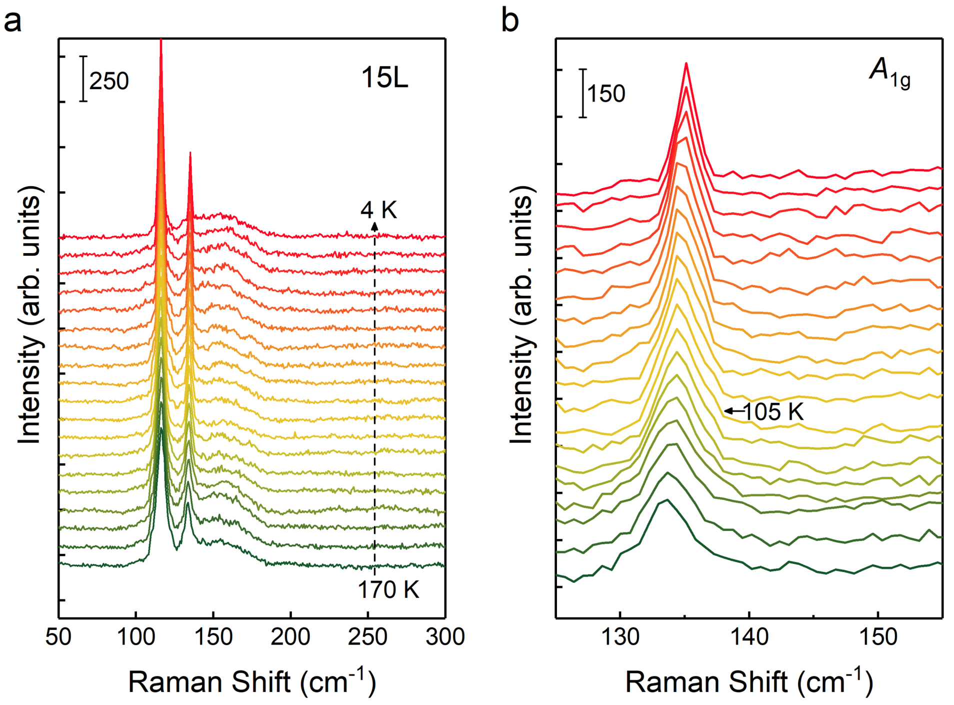

Supplementary Figure 10 | Temperature dependent Raman spectrum of 15L sample. a The Raman spectrum for 45L sample at temperature varied from 170 K to 4 K. Only two main lattice peak emerge. b Detail of evolution of the A_{1g} mode. The width of the A_{1g} mode evolves smoothly. Additional phonon modes near 150 cm^{-1} appears as a broad hump in spectrum of 15L sample which has also been reported in the previous Raman study. So far, the origin is unclear. It may be caused by some unknown surface modes.

Supplementary Note 7: Group analysis and CDW-induced modes

From the group-theoretical considerations, in the high temperature phase, phonon modes at the Γ-point of CsV_3Sb_5 are expressed as $\Gamma_{\text{total}} = A_{1g} + 4A_{2u} + B_{1g} + B_{1u} + 2B_{2u} + 2E_{2u} + E_{2g} + 5E_{1u} + E_{1g}$. Raman-active modes are $\Gamma_{\text{Raman}} = A_{1g} + E_{2g} + E_{1g}$. In the back-scattering geometry used in our experiment, the A_{1g} and E_{2g} can be detected in the *ab*-plane measurement, while the E_{1g} phonon can only be detected from the *ac* surface.

In the CDW phase, the structure is extending to a $2 \times 2 \times 1$ supercell, including two types of structures: Star of David (SoD) and inverse Star of David (iSoD). The space group of these two superlattices is $P6/mmm$, same as the high-temperature phase. Phonon modes at the Γ-point can be expressed as $\Gamma_{\text{total}} = 5A_{1g} + A_{1u} + 3A_{2g} + 9A_{2u} + 4B_{1g} + 5B_{1u} + 2B_{2g} + 7B_{2u} + 8E_{2u} + 8E_{2g} + 14E_{1u} + 6E_{1g}$. Raman-active modes are $\Gamma_{\text{Raman}} = 5A_{1g} + 8E_{2g} + 6E_{1g}$. Clearly, compared to the high-temperature phase, new CDW-induced folding modes will emerge.

Supplementary Fig. 11a shows the Raman spectrum of bulk with 1800 s exposure time. Our temperature-dependent Raman scattering measurements involve both high-temperature and low-temperature phases. At 100 K, only two Raman modes (A_{1g}, E_{2g}) can be observed for both bulk and flake samples, consistent with the group analysis mentioned above and the previous study[3]. At 4 K, seven new folding/amplitude modes emerge and all of them can be observed in the bulk[3,4]. To trace the CDW-induced folding modes in thin flakes, we extend the acquisition time for each round of measurement to 1 hour (3600 s), and repeat the measurement multiple times, taking the average to enhance the signal-to-noise ratio. We then scale the spectra to the highest phonon mode (E_{2g}) at 117 cm^{-1}, and all data are presented in $cnts \cdot mw^{-1} \cdot s^{-1}$ unit, as shown in Supplementary Fig. 12.

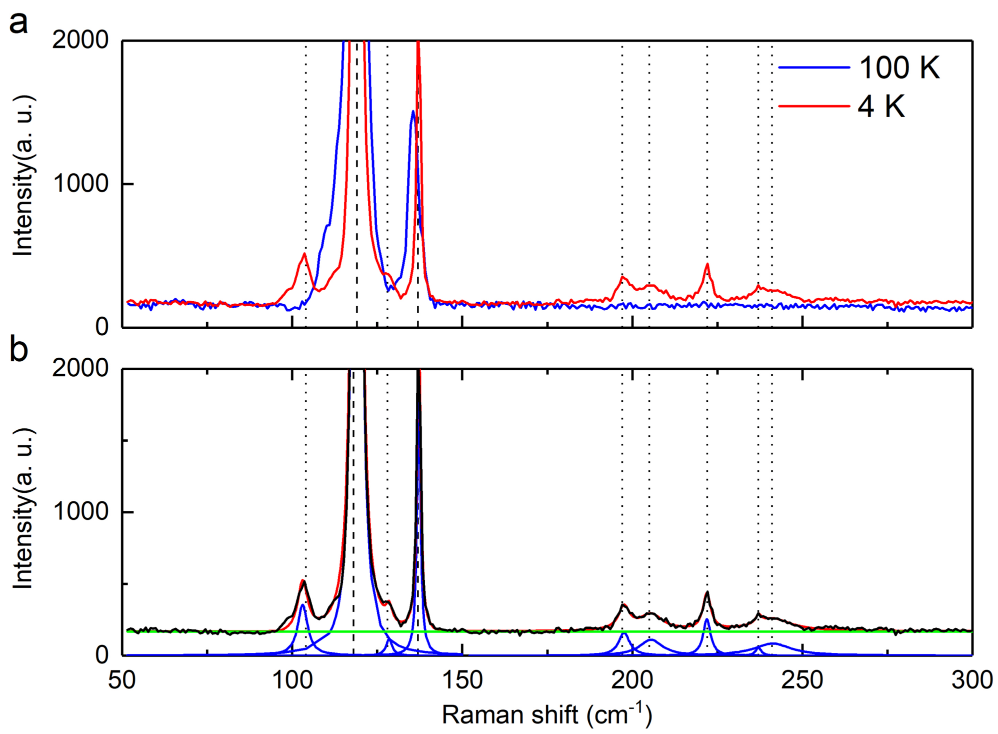

Supplementary Figure 11 | CDW-induced modes in bulk CsV_3Sb_5. a XX configuration measurement in bulk CsV_3Sb_5. Dashed lines show the main lattice modes. Dotted lines show CDW induced modes. **b** Lorentz fitting of the 100 K Raman spectrum in **a**. The black, green, blue, red lines are raw data, baseline, fitted peaks, fitted line, respectively.

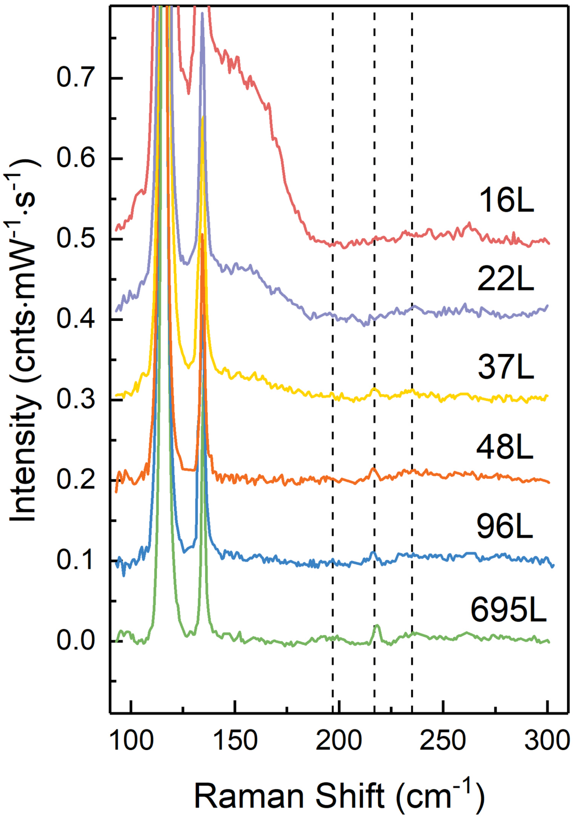

Supplementary Figure 12 | CDW induced modes in CsV_3Sb_5 thin flakes. Raman spectra of 695L, 96L, 48L, 37L, 22L, 16L thin flakes collected at 4 K. All the spectra have been scaled using the intensity of the E_{2g} phonon mode at 117 cm^{-1} and vertically shifted for clarity.

Supplementary Note 8: Anharmonic phonon decay model

We fit the temperature dependence of the phonon frequency and the FWHM using the anharmonic phonon decay model[5]:

$$\omega_1(T) = \omega_0 - C_1[1 + 2n(\Omega(T)/2)],$$

$$\Gamma_1(T) = \gamma_0 + \gamma_1[1 + 2n(\Omega(T)/2)],$$

where $\Omega(T) = \hbar\omega/k_BT$, $n(x) = 1/(e^x - 1)$ is the Bose-Einstein distribution function.

Supplementary Note 9: The sensitivity of A_{1g} mode

The intensity of the spectra is heavily dependent on the thickness of the exfoliated samples. To further identify the sensitivity of A_{1g} in flakes, we conducted a long-collection-time experiment for two flakes, 20L and 28L (Supplementary Fig. 13), covering the critical thickness of 25L with comparable peak intensity. The results show a clear frequency increment of A_{1g} frequency in the 28L sample across the CDW transition, but not in the 20L one.

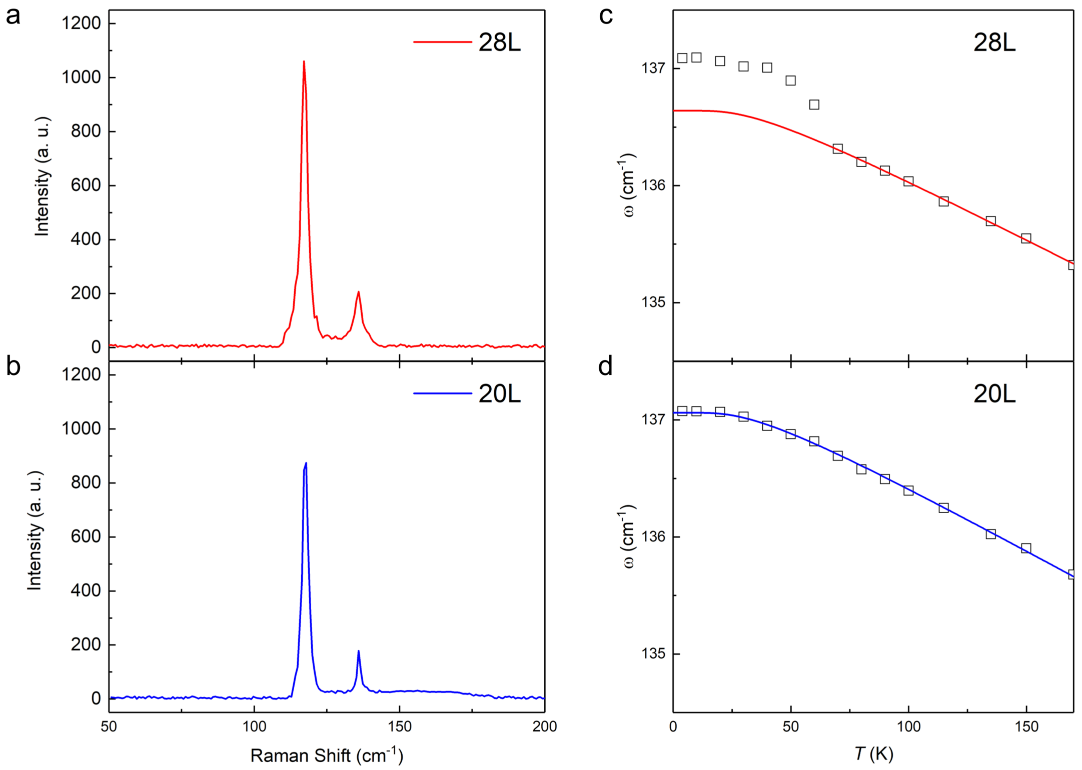

Supplementary Figure 13 | Temperature-dependent Raman response of 28L and 20L samples measured in the XX configuration. a, b Raman spectra of 28L and 20L collected at 4 K with comparable peak intensities. **c, d** Temperature-dependent evolution of the frequency of A1g mode for 28L and 20L samples.

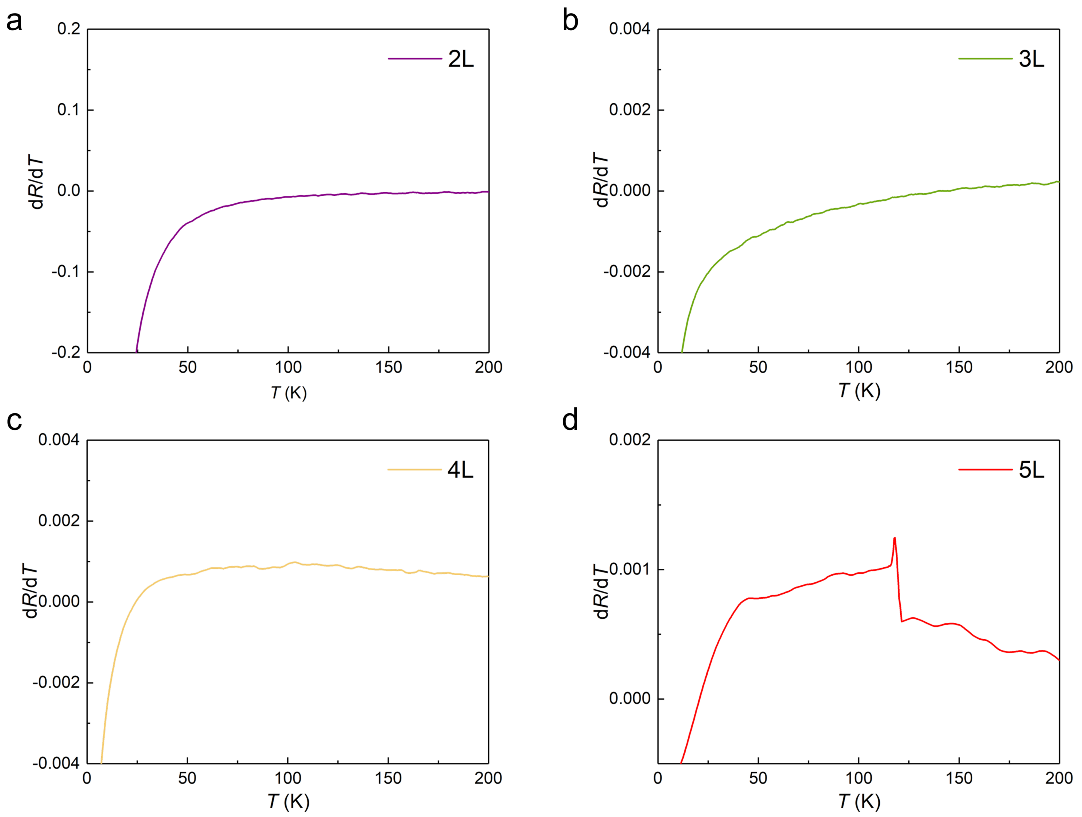

Supplementary Figure 14 | d*R*/d*T* plot of the 2-5L samples. No anomaly can be observed in the dR/dT plot of the 2 - 4L sample, which is in sharp contrast to the 5L sample.

Supplementary Note 10: Coulomb gap in atomically thin flakes

The non-metallic resistance in the 2L sample cannot be fitted by a band gap model, but follows a 2D variable-range-hopping[6] behavior at the temperature range 4-40 K shown in Fig. 4a of the main text. Supplementary Fig. 15 shows the fitting of the resistance below 3 K using the Efros-Shklovskii variable-range-hopping model[7]. The linear fit of the low-temperature region indicates the opening of a Coulomb gap induced by enhanced repulsive interaction. It can be understood as a direct consequence of dimension reduction with reduced carrier concentration, which eventually drives the MIT.

To determine if the MIT observed below 4L is an intrinsic property or a result of crystalline degradation during the Al_2O_3-assisted exfoliation process, we prepare a 16L sample with a T_{CDW} of 101.5 K using the Al_2O_3 method (Supplementary Fig. 16a).

After measuring its resistance, we further evaporate another layer of Al_2O_3 on the top of the device and measure its resistance again (Supplementary Fig. 16b). If the top few layers, say 4 layers, are degraded, the sample should behave like an 12L sample and the T_{CDW} will be enhanced to 110 K. However, as shown in Supplementary Fig. 16c, d, the T_{CDW} remains almost invariant, indicating that the kagome layers beneath the surface are less influenced by the Al_2O_3 deposition. Therefore, we believe that the observed insulating behavior approaching the atomic limit is intrinsic, rather than derived from the degradation.

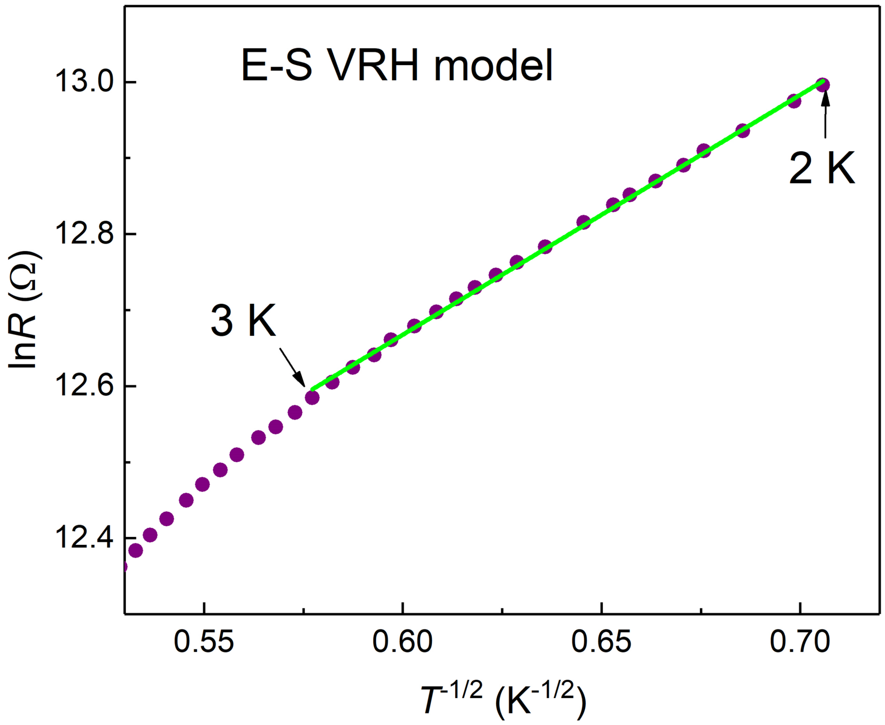

Supplementary Figure 15 | E-S VRH model fitting of the 2L sample. The resistance of the 2L sample below 3 K deviates from the 2D VRH model. But it can be fitted by the E-S VRH model which is a hallmark of the opening of a coulomb gap.

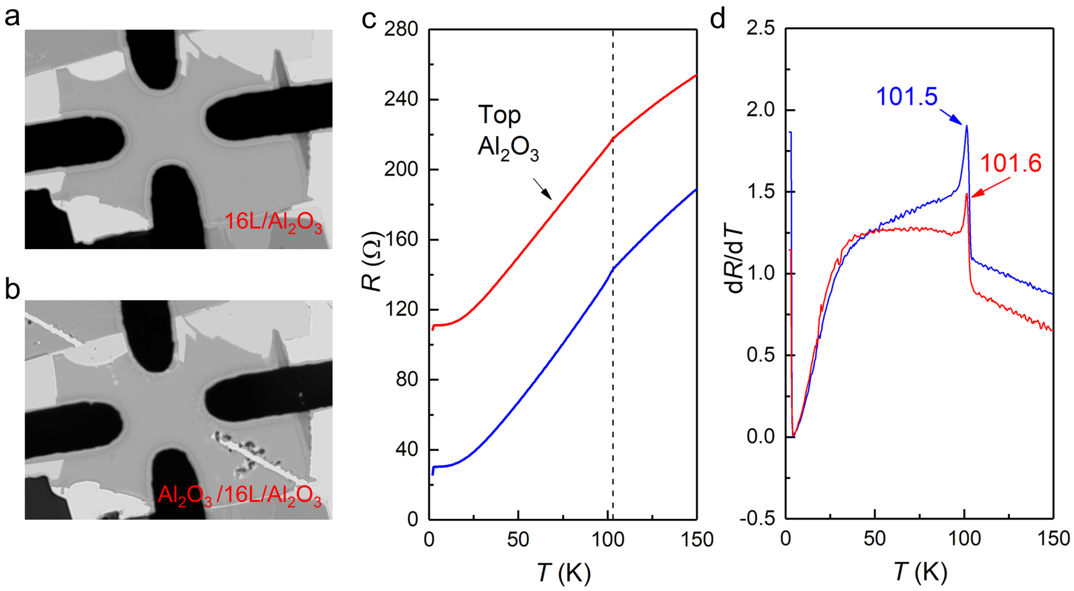

Supplementary Figure 16 | Surviving of surface sample proximity to Al_2O_3. a, b sample before and after top-Al_2O_3 deposition. **c, d** Resistance and first derivative dR/dT of the sample before (blue) and after (red) top-Al_2O_3 deposition with their T_{CDW}s at 101.5 K and 101.6 K, respectively.

Supplementary Note 11: Analysis of transport data

Supplementary Fig. 17a shows the raw resistance data of main text Fig. 2a. The low temperature resistance of samples in the CDW phase can be fitted use the power law equation $R = aT^{\alpha} + b$ in which the extracted α are around 3.4. The fitting works well with the thickness down to 12L. In 7L sample, the resistance below 4K slightly greater than fitted result, which is more distinct in thinner sample because of rerising of the resistance.

Supplementary Fig. 18a shows the normalized resistance of samples in normal metal phase without shift. Resistance in this regime can be linearly fitted. Supplementary Fig. 18b shows the extracted slope. The slope remains nearly constant with the reduction of sample thickness to ~25L, and then decrease drastically.

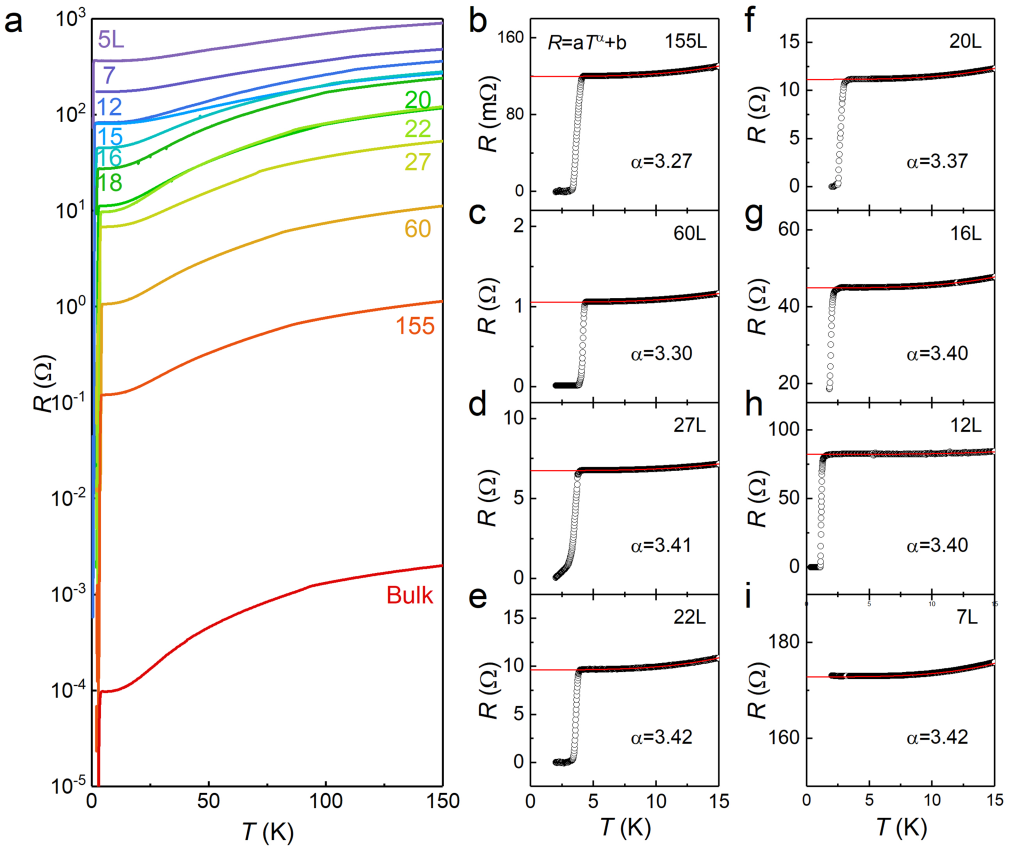

Supplementary Figure 17 | Resistance of CsV_3Sb_5 thin flakes. a Raw data of resistance without normalization or shifting. The thicknesses of samples are corresponding to the line with same color in main text Fig. 2a. **b-i** Power-law fitting of low-temperature resistance.

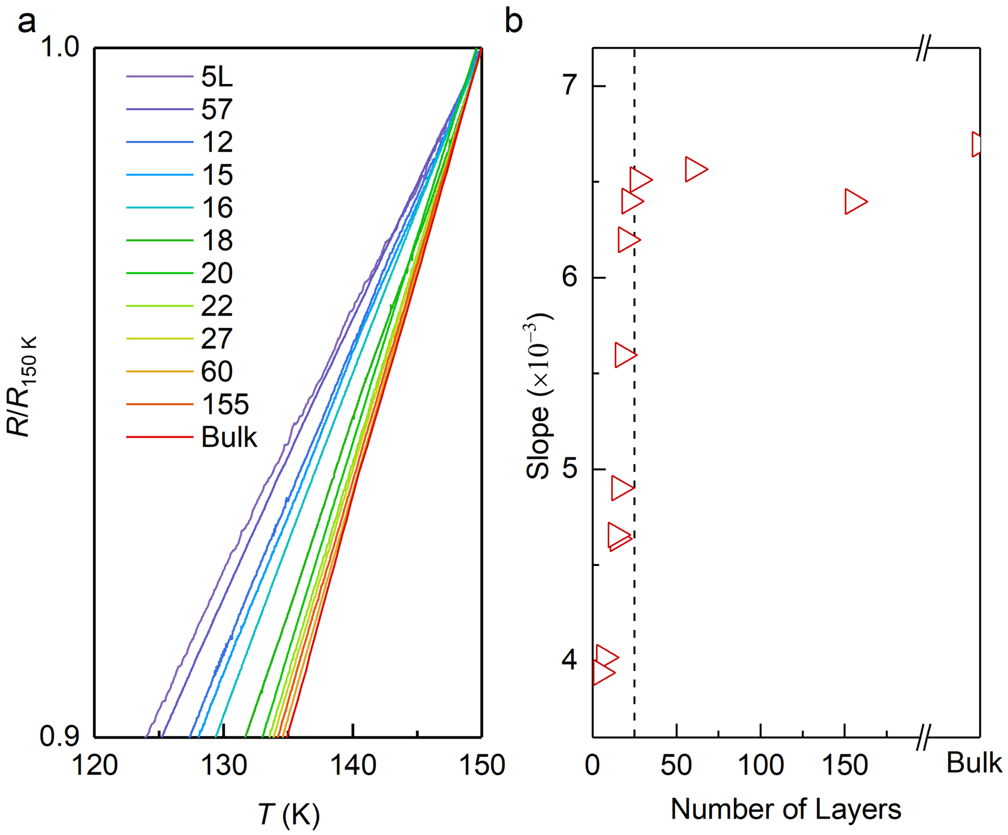

Supplementary Figure 18 | Linear fitting of resistance above T_{CDW}. a Normalized resistance of samples in normal metal phase. **b** Slope extracted from linear fitting of data in **a**. The dashed line indicates the critical thickness of 25L.

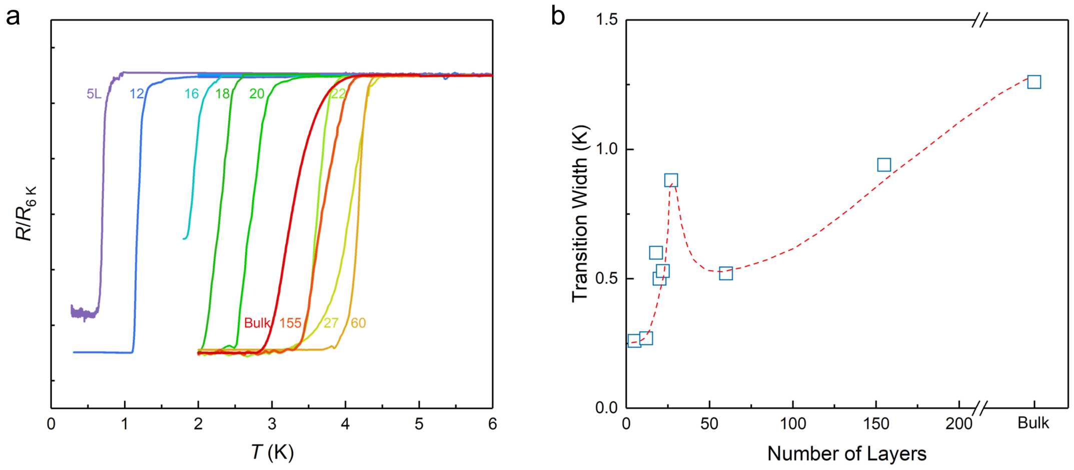

Supplementary Figure 19 | Superconducting transition width. Scaled low-temperature resistance and extracted superconducting transition width as a function of sample thickness.

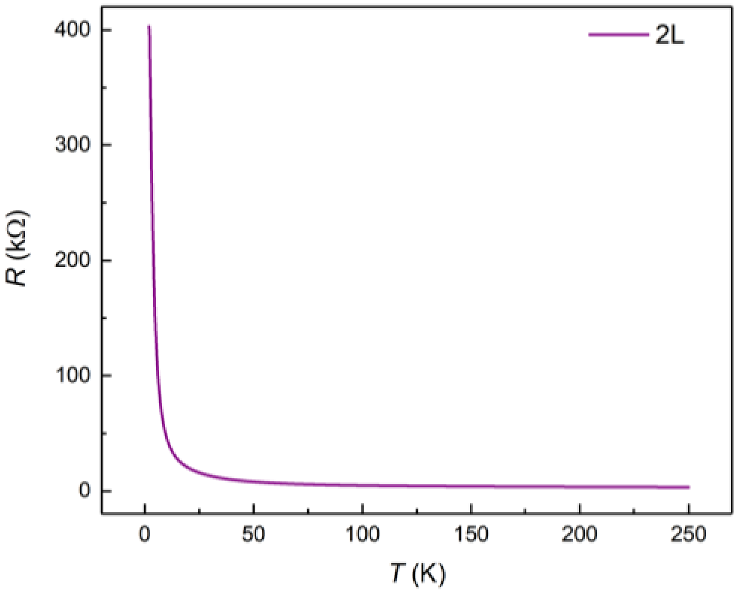

Supplementary Figure 20 | Raw data of 2L sample from 2-250 K. The maximum resistance is 0.4 MΩ at 2 K. The resistance at 2 K remained almost constant under different excitation currents (1 μA, 20 μA), indicating that the contact heating effect is negligible.

Supplementary References

1. Zhao, H. et al. Cascade of correlated electron states in the Kagome superconductor CsV_3Sb_5. *Nature* **599**, 216–221 (2021).
2. Song, Y. et al. Competition of Superconductivity and Charge Density Wave in Selective Oxidized CsV_3Sb_5 Thin Flakes. *Phys. Rev. Lett.* **127**, 237001 (2021).
3. Liu, G. et al. Observation of anomalous amplitude modes in the kagome metal CsV_3Sb_5. *Nat. Commun.* **13**, 3461 (2022).
4. Wu, S. et al. Charge density wave order in the kagome metal AV_3Sb_5 (A=Cs, Rb, K). *Phys. Rev. B* **105**, 155106 (2022).

5. Klemens, P. G. Anharmonic Decay of Optical Phonons. *Phys. Rev.* **148**, 845–848 (1966).
6. Mott, N. F. Conduction in glasses containing transition metal ions. *J. Non-cryst. Solids* **1**, 1–17 (1968).
7. Efros, A. L. & Shklovskii, B. I. Coulomb gap and low temperature conductivity of disordered systems. *J. Phys. C: Solid State Phys.* **8**, L49 (1975).
